# A Novel Convolutional Neural Network–Based Algorithm for Heart Rate Measurement From Ballistocardiography Signals in Diverse Clinical Settings: Observational Study

**DOI:** 10.2196/71224

**Published:** 2026-07-30

**Authors:** Kumar Chokalingam, Muthukumarasamy Saravanan, Ashish Kaushal, Srishti Rao, Inam Ur Rahman, Ashwathi Nambiar, Mudit Dandwate, Ravi Mahajan, Kunal Sarkar, Gaurav Parchani

**Affiliations:** 1 Department of Clinical Research, Turtle Shell Technologies Private Limited, Ground and Mezzanine Floor, Nomads Daily Huddle, City Centre, 40, Chinmaya Mission Hospital Road, Stage 2, Hoysala Nagar, Indiranagar, Bengaluru, 560038, India, 91 9008349922

**Keywords:** heart rate measurement, ballistocardiography, convolutional neural networks, contactless monitoring, health care technology, signal processing, remote patient monitoring

## Abstract

**Background:**

Continuous vital sign monitoring ensures early detection, prevents intensive care unit (ICU) admissions, and improves patient outcomes. Continuous heart rate (HR) monitoring methods often require direct skin contact, which can lead to patient discomfort. The rising popularity of ballistocardiography (BCG) offers a promising, noncontact solution for continuous vital sign monitoring with improved patient comfort.

**Objective:**

This study aims to develop and validate a novel HR measurement algorithm leveraging convolutional neural networks (CNNs) and BCG signals for accurate, noncontact, and continuous HR monitoring. By integrating time-domain peak detection with short-time Fourier transform and CNN models, the proposed approach seeks to enhance HR measurement accuracy across diverse health care settings. The study follows the Food and Drug Administration (FDA)’s Good Machine Learning Practice guidelines and evaluates the algorithm’s robustness, generalizability, and clinical applicability through extensive testing on a diverse dataset, ensuring improved patient comfort and early detection of clinical deterioration.

**Methods:**

The proposed algorithm combines time-domain peak detection with short-time Fourier transform and CNNs to enhance HR measurement from BCG signals. The CNN model developed was trained on 129,976 data points from 373 participants (HR range: 36‐230 bpm), including ICU patients, and was tuned on 75,970 data points from 192 participants (HR range: 46‐169 bpm), with HR obtained from clinical-grade electrocardiography devices to improve generalizability. The algorithm was tested on 70,211 data points from 205 participants, including ICU patients, across 5 independent studies to demonstrate robust performance against diverse settings, demographics, and comorbidities. The methodology is in compliance with the FDA’s *Good Machine Learning Practice for Medical Device Development: Guiding Principles*.

**Results:**

The algorithm achieved a mean absolute error of under 3 bpm and a detection rate exceeding 80%, underscoring its robustness. The Bland-Altman analysis indicates high accuracy with a minimal bias of 0.25 and limits of agreement within 8.59 bpm. Additionally, the Pearson correlation coefficient of 0.97 from the Deming regression further demonstrates strong alignment with reference HR measurements, reinforcing its precision and reliability for clinical applications.

**Conclusions:**

This CNN-based algorithm presents a robust solution for contactless HR monitoring, addressing the limitations of prior methods in noise management and adaptability. Its demonstrated accuracy, particularly in real-world, noisy clinical environments, highlights its potential for broad application in patient monitoring and improved comfort.

## Introduction

Continuous monitoring of heart rate (HR), respiratory rate, and blood pressure in ward patients enables early detection of deterioration, reducing unplanned intensive care unit (ICU) admissions and rapid response activations while improving outcomes [1-4]. Current continuous HR monitoring techniques, such as electrocardiography (ECG) [5] and photoplethysmography [6,7], require direct skin contact, which can cause discomfort and increase the risk of irritation during prolonged use [8,9]. This limitation is particularly relevant for patients requiring continuous monitoring, where skin-contact sensors may further complicate patient care and comfort. As health care trends shift toward noninvasive solutions, there is a growing demand for continuous, contactless HR monitoring technologies that ensure patient comfort without compromising accuracy.

**Figure 1.**
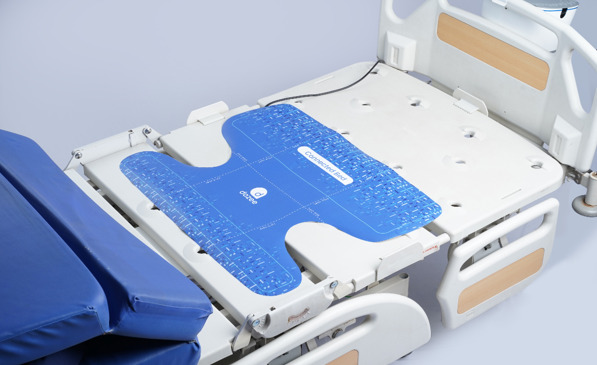
A sensor sheet positioned beneath the bed captures subtle microvibrations from the patient, enabling noninvasive measurement of vital signs.

Ballistocardiography (BCG), which captures the body’s micromovements induced by cardiac contractions, has emerged as a promising contactless technique for monitoring cardiovascular activity. By detecting mechanical vibrations generated by the heart’s pumping action, BCG offers a noninvasive means of measuring HR [10-12]. This makes it particularly suitable for long-term monitoring in settings such as older adult care, postoperative recovery, and remote patient care, where minimizing discomfort and maximizing patient compliance are essential [13]. Dozee (Turtle Shell Technologies Pvt Ltd) has successfully integrated BCG technology with advanced algorithms to develop a remote patient monitoring system capable of continuously tracking HR, respiratory rate, and sleep patterns [3,14-19]. This system uses a sensor sheet positioned beneath the mattress (Figure 1) to capture vital signs without direct patient contact, providing an unobtrusive solution that is ideal for long-term use in various health care environments.

Despite its potential, HR measurement from BCG signals remains challenging due to noise, motion artifacts, and physiological variability, which can degrade signal quality and compromise accuracy [20-23]. Reliable HR measurement requires isolating cardiac components from a composite signal that also includes respiratory information, body movement information, and environmental noise. To achieve this, BCG signal processing often uses band-pass filtering or advanced decomposition techniques to separate signal components. Following signal separation, various algorithmic approaches—such as time-domain, frequency-domain, and wavelet-transform methods—can be applied to extract meaningful HR metrics [24].

Previous BCG-based HR estimation methods relied heavily on traditional signal processing techniques, such as peak detection in the time domain. For instance, Dozee earlier approaches used time-domain peak detection, which demonstrated good performance in controlled conditions, with HR measurements typically within 3 bpm of reference values [15,18,19]. However, this accuracy diminished in real-world environments due to noise and body movement, limiting the effectiveness of these methods in clinical scenarios.

Recent advancements in machine learning (ML) and artificial intelligence have enabled the development of data-driven models that can recognize complex patterns in noisy datasets, providing significant improvements in HR measurement accuracy. These ML-based methods often require extensive datasets and substantial computational resources. However, many current ML-based models are trained on limited datasets, which can hinder their ability to generalize effectively across diverse patient populations and clinical conditions [25,26].

In response to these limitations, we introduce a novel convolutional neural network (CNN)-based algorithm designed to improve HR estimation from BCG signals. This algorithm combines time-domain peak detection with frequency-domain analyses, leveraging CNNs to extract higher-order features from the BCG signal. By using both time- and frequency-domain information, our hybrid approach effectively addresses challenges related to noise and motion artifacts, offering enhanced accuracy in diverse and complex clinical environments.

Our algorithm was trained on a large, demographically diverse dataset encompassing various clinical and nonclinical environments to ensure robustness and generalizability. Following the practices laid down in the Food and Drug Administration (FDA)’s *Good Machine Learning Practice for Medical Device Development: Guiding Principles*, we tested the algorithm on a mutually exclusive dataset collected independently from the training and tuning data [27]. This rigorous validation underscores the algorithm’s potential for reliable, contactless HR monitoring across a wide range of health care applications, from hospital wards to remote patient monitoring [27].

## Methods

### Study Design

This retrospective analysis used previously collected BCG signals and reference HR data from multiple research studies, including those in which HR evaluation was either a primary or secondary objective.

### Ethical Considerations

For studies in which HR evaluation was not a primary or secondary focus, as well as for the overall retrospective analysis, ethical approval (ECG024/2024) was obtained from the Genebandhu Independent Ethics Committee, New Delhi, India, to ensure adherence to established ethical guidelines. Patient confidentiality was rigorously maintained through the anonymization of all data in compliance with data protection regulations. Furthermore, informed consent was obtained from all participants during the original data collection, upholding ethical standards and safeguarding participants’ rights. No compensation was provided to participants.

### Dataset Overview

The dataset includes BCG signals and reference HR measurements gathered from a range of clinical and nonclinical environments. In these studies, BCG and reference HR measurements were captured simultaneously, with HR data obtained from clinical-grade ECG devices. Reference HR values from clinical-grade ECG devices served as an accurate benchmark for evaluating the algorithm’s performance. BCG data were gathered using the Dozee contactless monitoring system. Data collection covered a range of conditions, including rest, movement, and sleep. All reference ECG-derived HR values underwent quality screening to remove physiologically implausible readings or artifact-induced errors. Spurious values (eg, HR<20 or >250 bpm, or sudden changes >50% within 1 measurement interval) were flagged and excluded. By filtering out these outliers and any HR readings noted as affected by clinical interventions, we ensured that the reference standard was reliable for algorithm training and validation.

For the algorithm’s development and validation, the dataset was segmented into 3 subsets—training, tuning, and testing—reflecting different stages of the algorithm’s development. These subsets encompass a diverse participant pool, ensuring representation across various age groups, sex, and health conditions.

Notably, the training or tuning and testing sets were defined a priori by separate clinical researchers, and the data scientists developing the model were blinded to the allocation. The training or tuning and testing partitions were selected to ensure broad representation across HR ranges, comorbidities, demographics, and participant characteristics. This strict separation and blinding process followed recommended practices, eliminating any risk of data leakage between training and validation cohorts and ensuring an unbiased performance evaluation.

### Training and Tuning Datasets

**Figure 2.**
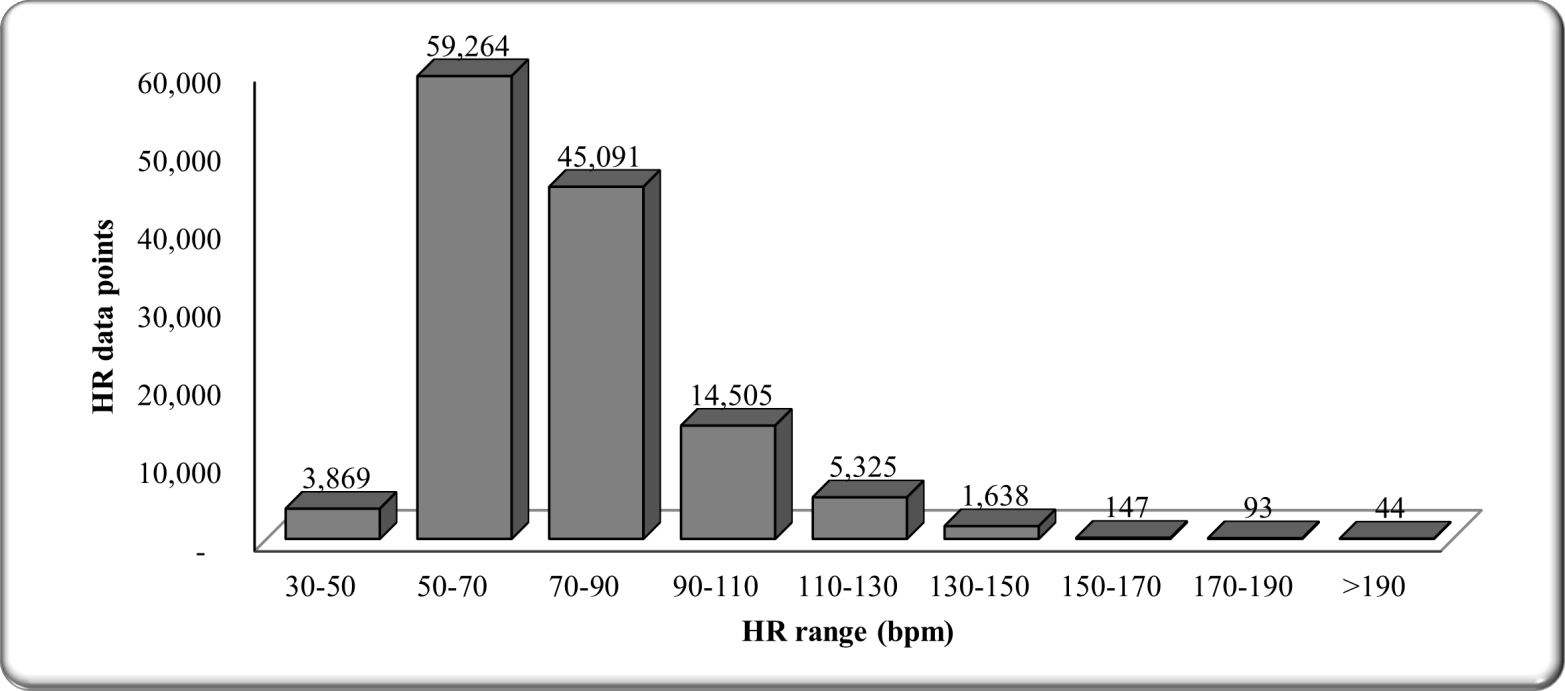
Heart rate (HR) data points across different ranges in the (A) training dataset and (B) tuning dataset showcasing a diverse representation across all ranges.

**Figure 3.**
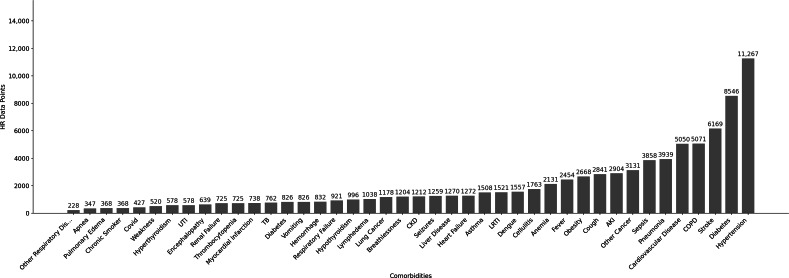
Heart rate (HR) data points across comorbidities in the (A) training dataset and (B) tuning dataset, showcasing a diverse representation of health conditions. AKI: acute kidney injury; CKD: chronic kidney disease; COPD: chronic obstructive pulmonary disease; LRTI: lower respiratory tract infection; TB: tuberculosis; UTI: urinary tract infection.

The training and tuning datasets were drawn from 10 studies conducted at 6 locations across India (Table 1), covering both clinical and home settings to support the comprehensive development of the algorithm across diverse environments and patient demographics. The training dataset included data from 373 participants, totaling 129,976 data points (Table 2). This dataset was used to train a CNN to recognize HR patterns within BCG signals, covering an HR range of 36 to 230 bpmTable 2 and Figure 2A) and a varied range of comorbidities (Figure 3A). This extensive range supports the algorithm’s robustness across varied cardiac activities and demographics.

**Table 1.** Overview of study locations and participant populations for the training and tuning dataset.

Location	Number of studies	Study participants
1. Apollo Hospitals, Jubilee Hills, India	3	ICU^a^ and ward patients
2. Sparsh Hospitals, Bengaluru, India	2	Healthy volunteers and ward patients
3. Jayadeva Institute of Cardiovascular Sciences and Research, Bengaluru, India	1	Ward patients
4. National Institute of Mental Health and Neurosciences, Bengaluru, India	2	Healthy volunteers
5. Indira Gandhi Institute of Medical Sciences, Patna, India	1	ICU patients
6. Dozee and Home Settings, Bengaluru, India	1	Healthy volunteers

a ICU: intensive care unit.

**Table 2.** Summary of demographic and heart rate (HR) data across datasets.

	Training	Tuning	Combined
Subjects	373	192	565
Sex (male:female)	242:128^a^	116:70^c^	358:198^e^
Age (y), mean (SD; range)	38^b^ (16.83; 19-90)	37^c^ (16.36; 19-88)	38^f^ (16.66; 19-90)
Weight (kg), mean (SD; range)	69.16^b^ (14.98; 35-125)	71.18^d^ (16.22; 40-135)	69.83^g^ (15.42; 35-135)
Height (m), mean (SD; range)	1.67^b^ (0.10; 1.25-1.87)	1.68^e^ (0.09; 1.49-1.85)	1.67^h^ (0.10; 1.25-1.87)
BMI, mean (SD; range)	24.86^b^ (4.93; 14.17-48)	25.27^e^ (4.85; 16.22-43.70)	24.99^h^ (4.90; 14.17-48)
HR data points	129,976	75,970	205,946
HR range	36-230	46-169	36-230

a = 3

b = 5

c = 6

d = 7

e = 9

f = 11

g = 12

h = 14

The tuning dataset included 75,970 data points from 192 participants Table 2 and was used to refine the algorithm’s parameters for optimal performance. This subset facilitated adjustments to the CNN’s architecture and hyperparameters, enhancing the algorithm’s ability to generalize beyond the training data. The HR range within this tuning dataset, 46 to 169 bpm (Table 2 and Figure 2B), reflects typical cardiac activity in the general population, and the inclusion of a varied range of comorbidities (Figure 3B) further strengthens the algorithm’s applicability across diverse user groups.

### Testing Dataset

**Figure 4.**
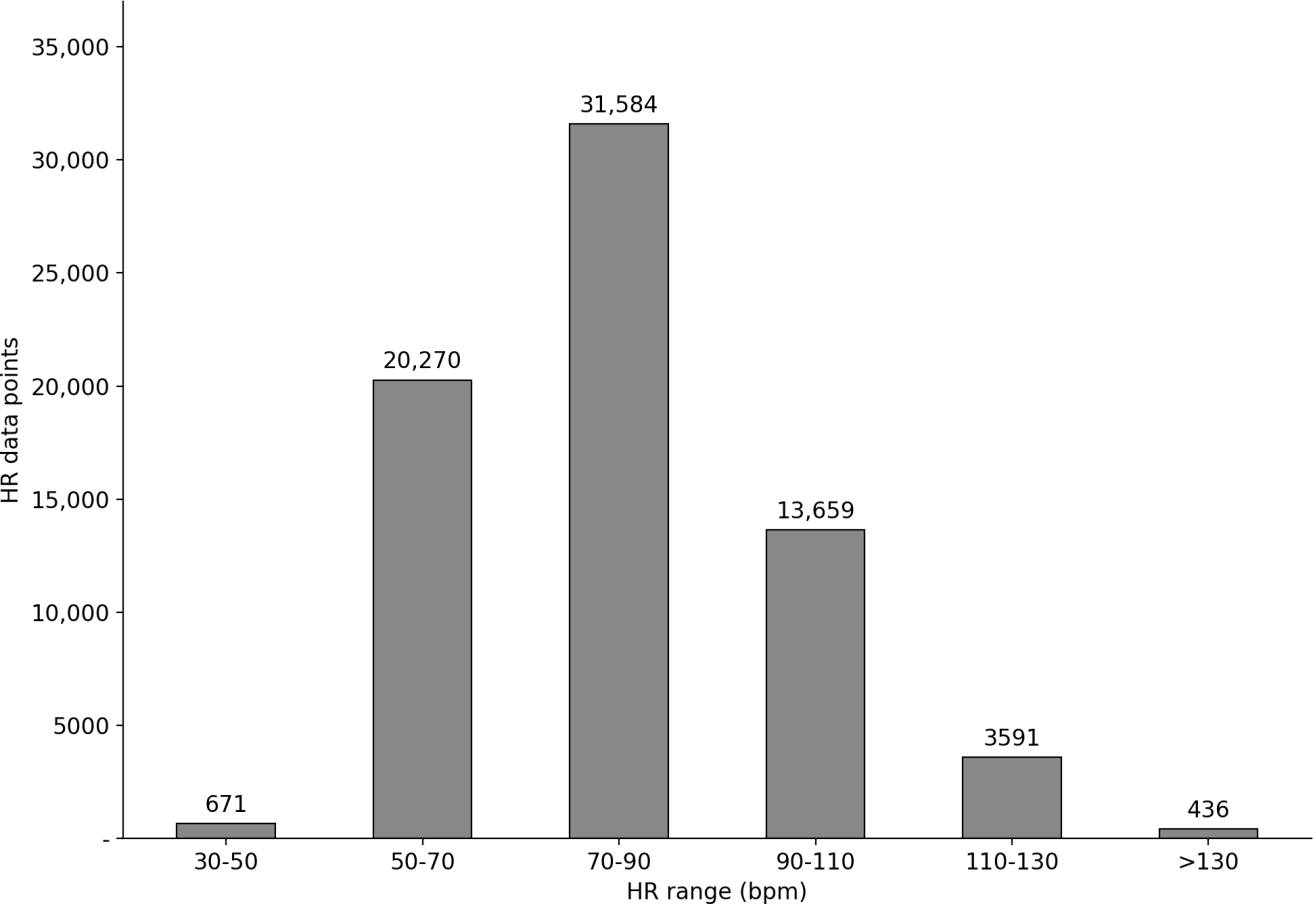
Heart rate (HR) data points across different ranges in the testing dataset, showcasing a diverse representation across all ranges.

**Figure 5.**
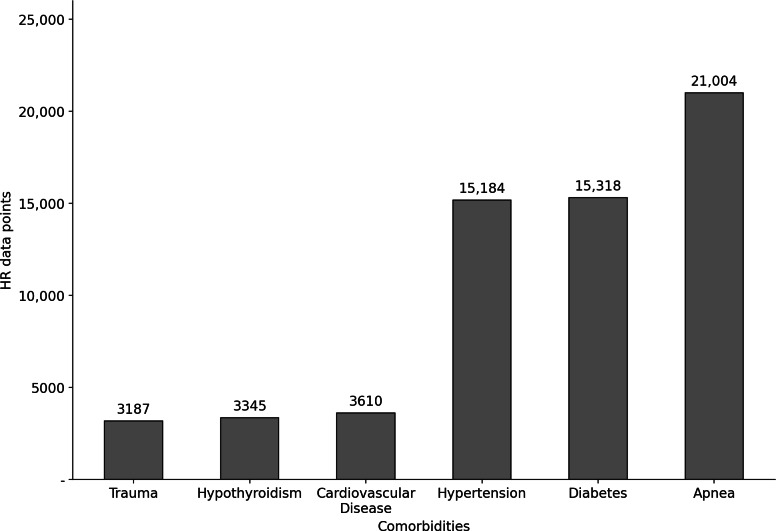
Heart rate (HR) data distribution across comorbidities, each with at least 3000 data points.

The testing dataset was sourced from 5 independent clinical studies conducted at various locations (Table 3), entirely separate from the training and tuning data to ensure unbiased evaluation and minimize the risk of overfitting. This dataset included data from 205 participants and 70,211 data points, covering an HR range of 40 to 169 bpm (Table 4 and Figure 4) and a range of comorbidities, each with at least 3000 data points (Figure 5). The diversity in participant demographics, including different age groups, sex, and health conditions, provided a robust foundation for evaluating the algorithm’s performance across a wide range of patient populations and clinical environments.

**Table 3.** Overview of study locations and participant populations for the testing dataset.

Study	Study title	Location	Study participants
Study 1	Pilot Clinical Evaluation of Dozee VS in Hospital Patients	Eastside Research Centre, Seattle, United States	Healthy volunteers
Study 2	Evaluation of Continuous Non-Invasive Blood Pressure Monitoring through Dozee	BGS Global Gleneagles Hospital, Bengaluru, India	ICU^a^ patients
Study 3	Dozee as a Screening Tool for Sleep Apnea Detection and Classification	St. John’s Medical College Hospital, Bengaluru, India	Patients with apnea
Study 4	Dozee as a Screening Tool for Sleep Apnea Detection and Classification	Apollo Hospital, Bannerghatta Road, Bengaluru, India	Patients with apnea
Study 5	Dozee as a Screening Tool for Sleep Apnea Detection and Classification	Nithra Institute of Sleep Sciences, Chennai, India	Patients with apnea

a ICU: intensive care unit.

**Table 4.** Overview of demographics and heart rate (HR) for the testing dataset.

Characteristics	Study 1 (n=50)	Study 2 (n=71)	Study 3 (n=25)	Study 4 (n=53)	Study 5 (n=6)	Combined (n=205)
Sex (male:female)	12:38	47:23^a^	18:7	32:21	6:0	115:89^a^
Age (y), mean (SD; range)	33 (13.74; 18-58)	53 (17.19; 20-94)^a^	49 (15.19; 19-84)^a^	54 (14.47; 18-90)	45 (12.94; 27-59)	48 (17.47; 18-94)^a^
Weight (kg), mean (SD; range)	72.48 (15.85; 46-116)	69.39 (11.92; 50-115)^a^	81.19 (12.94; 55‐103.5)^a^	86.28 (15.57; 59-132)	90.63 (27.29; 65-131)	76.58 (16.26; 46-132)^a^
Height (m), mean (SD; range)	1.68 (0.11; 1.49‐1.93)	1.67 (0.06; 1.44‐1.75)^a^	1.63 (0.08; 1.52‐1.80)^a^	1.63 (0.10; 1.40‐1.85)	1.70 (0.08; 1.59‐1.79)	1.66 (0.09; 1.40‐1.93)^a^
BMI, mean (SD; range)	25.52 (4.58, 18‐35.81)	24.88 (4.90; 19.53‐48.23)^a^	30.64 (5.56; 19.86‐40.82)^a^	32.47 (4.72; 23.1‐45.1)	30.73 (6.75; 23.9‐41.8)	27.88 (5.90; 18‐48.23)^a^
HR data points	6086	27,194	11,189	23,263	2479	70,211
HR range	40‐108	44‐169	42‐128	48‐119	46‐112	40‐169

a Depicts the respective participants’ missing data.

### Preprocessing of BCG Signals

#### Overview

**Figure 6.**
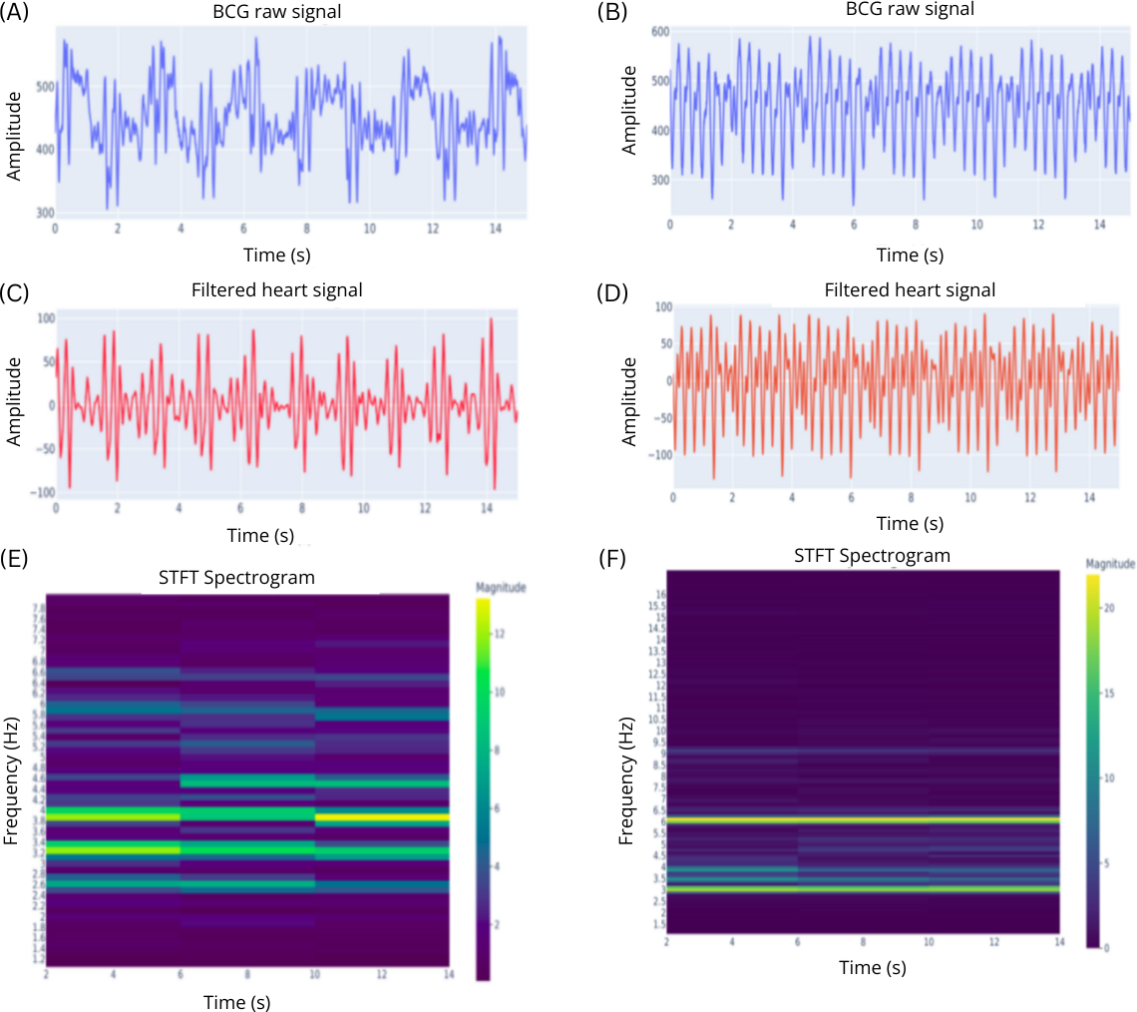
(A) & (B): Raw ballistocardiography (BCG) signals showing cardiac contractions and respiration. Filtered cardiac signals: (C) 9.5 beats in 15 seconds (38 bpm; reference 39 bpm) and (D) 46 beats in 15 seconds (182 bpm; reference 182 bpm). (E) & (F): Spectrograms highlighting the dominant cardiac frequencies for each segment. STFT: short-time Fourier transform.

Preprocessing steps were applied to BCG raw signals (Figure 6A and B, top panel) to reduce noise and artifacts introduced by movement and other physiological factors, ensuring a clean input for the HR measurement algorithm. Each 2-minute BCG recording was subsequently segmented into shorter epochs corresponding to periods of patient stillness. Segments contaminated by movement artifacts or excessive noise were automatically discarded, ensuring that only high-quality, artifact-free signal portions were used for HR analysis.

#### Artifact Removal

Motion artifacts were detected using adaptive filtering techniques, and corrupted signal segments were excluded from further analysis to improve the accuracy of HR measurement.

#### Noise Reduction

A band-pass filter of 0.5 to 25 Hz was applied to remove the low- and high-frequency noises and the respiration of BCG signals (Figure 6A and B, middle panel).

### HR Measurement Algorithm

The HR measurement algorithm was designed to extract fundamental and harmonic frequency components from the BCG signal, combining signal processing techniques with a CNN for robust HR detection under diverse conditions. Figure 6 illustrates the algorithm’s process on a sample segment: the raw BCG waveform and its filtered version, alongside the short-term Fourier transform (STFT) spectrogram. In these examples, the algorithm correctly identifies an HR of approximately 39 bpm and approximately 182 bpm from BCG signals, closely matching the reference ECG. The HR measurement algorithm includes the following processes:

- Heart signal fundamental and harmonic frequencies extraction: a STFT was applied to the filtered signal segments, generating a spectrogram in which the periodic nature of heartbeats manifested as fundamental and harmonic frequency components. These frequencies, indicative of the regularity of heartbeats, were used to distinguish and infer the HR from other noises. For example, if the harmonic spacing corresponded to a fundamental frequency of 3.03 Hz, this translates to an HR of approximately 182 bpm (Figure 6A and B bottom panel).
- A CNN algorithm was trained with both BCG signals and spectrograms as input. The CNN learns to recognize HR patterns from the BCG signals and frequency components within the spectrogram. During inference, the CNN detects fundamental frequencies and counts heartbeats from BCG signals, providing accurate HR measurements even under challenging conditions such as noise or motion artifacts. The CNN architecture was specifically designed to handle complex patterns in BCG signals and their spectrograms, achieving high accuracy by simultaneously analyzing both frequency and time domains.
- Adaptive filtering: the approach uses an iterative, dynamically adjusted thresholding mechanism that continuously evaluates real-time signal quality, enabling the system to automatically refine its sensitivity and thresholds to distinguish genuine cardiac signals from transient artifacts and environmental noise.

### Algorithm Evaluation

The performance of the HR measurement algorithm was assessed on the testing dataset by comparing measured HR values to reference values using the following metrics to evaluate accuracy and agreement:

- Mean absolute error (MAE): the average absolute difference between the measured and reference HR values was calculated, providing an indicator of the algorithm’s overall accuracy.
- Detection rate (DR): the percentage of time the algorithm successfully detected and measured HR from BCG signals was evaluated to assess robustness in real-world clinical environments where signal quality may vary.
- Bland-Altman: this method was used to analyze the agreement between measured and reference HR values, identifying any systematic bias by examining the distribution of differences.
- Deming regression with the Pearson correlation coefficient: this regression method, which accounts for measurement errors in both variables, was used to evaluate the alignment between measured and reference HR values. The Pearson correlation coefficient was calculated to quantify the strength and direction of the linear relationship between the algorithm’s HR measurements and clinical reference values.

These metrics were chosen to facilitate comparison with prior studies. MAE, Bland-Altman limits of agreement, correlation coefficients, and detection rates are standard benchmarks for validating unobtrusive HR estimation algorithms [28-34]. Using this common set of metrics allows us to contextualize our results against existing BCG-based and other noncontact HR monitoring approaches.

These evaluation metrics provide a comprehensive analysis of the algorithm’s accuracy, reliability, and applicability in real-world clinical settings, reinforcing its potential as a contactless solution for HR monitoring.

## Results

### MAE and DR Analysis

The HR measurement algorithm demonstrated high accuracy and reliability across datasets from studies conducted in both the United States and India. In total, the algorithm processed 70,211 reference points and produced 58,908 measured points, resulting in an MAE of 2.50 bpm and a DR of 83.90%.

In the US-based study 1, the algorithm achieved an MAE of 2.27 bpm and a DR of 92.34%. The algorithm’s performance remained robust in the India-based studies (studies 2‐5), with a combined MAE of 2.52 bpm and a DR of 83.10%. Specifically, study 2 reported an MAE of 2.73 bpm and a DR of 81.40%, while study 3 achieved an MAE of 2.49 bpm and a DR of 81.12%. Study 4 achieved an MAE of 2.39 bpm and a DR of 84.52%. Notably, study 5 demonstrated the best performance, with an MAE of 1.78 bpm and a DR of 97.26%.

These findings highlight the algorithm’s consistency across diverse geographic locations and clinical environments, maintaining an MAE below 3 bpm and a DR exceeding 80% across all datasets. Table 5 provides a detailed summary of the performance metrics from the five studies, offering a comprehensive overview of the algorithm’s accuracy and detection capabilities.

**Table 5.** Summary of heart rate (HR) measurement algorithm performance across 5 studies.

Variables		Reference data points	Measured data points	MAE^a^	DR^b^
Total		70,211	58,908	2.50	83.90
Study wise					
Study 1		6086	5620	2.27	92.34
Study 2		27,194	22,137	2.73	81.40
Study 3		11,189	9077	2.49	81.12
Study 4		23,263	19,663	2.39	84.52
Study 5		2479	2411	1.78	97.26
Study location					
The United States		6086	5620	2.27	92.34
India		64,125	53,288	2.52	83.10

a MAE: mean absolute error.

b DR: detection rate.

**Figure 7.**
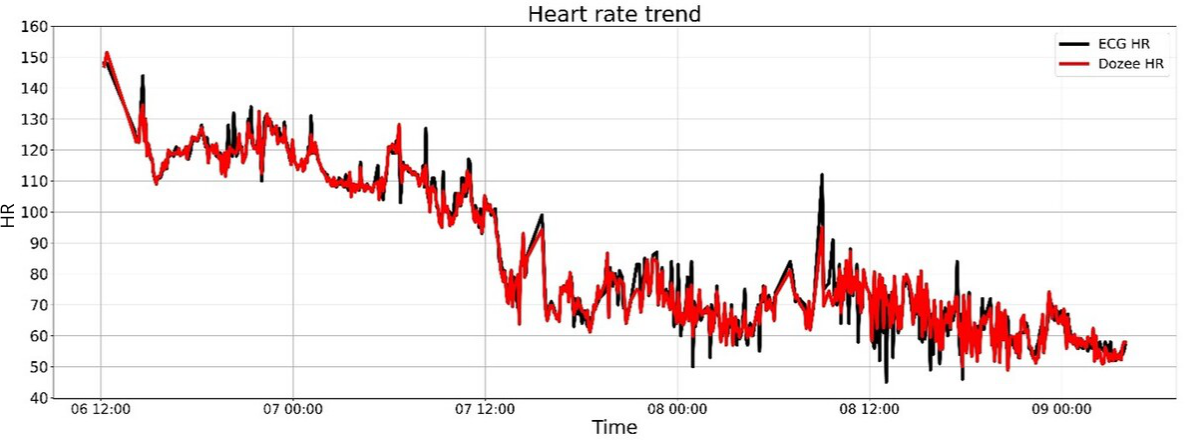
Heart rate (HR) trend for an intensive care unit (ICU) patient, comparing HR values measured by the convolutional neural networks (CNNs)-based algorithm with clinical-grade electrocardiography (ECG) reference HR measurements over several hours.

Figure 7 shows the HR trend for an ICU patient, comparing HR values measured by the CNN-based algorithm with clinical-grade ECG reference HR measurements over several hours. The figure illustrates that the HR measurement algorithm closely aligns with the ECG reference values, even as the patient’s HR fluctuates within a broad range of 50 to 150 bpm. The visual alignment between the Dozee HR measurements and the ECG HR reference across varying HR levels highlights the algorithm’s precision and responsiveness in a real-world clinical setting. This result demonstrates the algorithm’s capability to maintain accurate HR tracking in the high-noise ICU environment, underscoring its potential as a viable, contactless solution for real-time HR monitoring in health care settings.

A demographic analysis of the algorithm’s performance showed stable accuracy across a variety of subgroups, including different HR ranges, age groups, sex, BMI categories, racial backgrounds, and comorbidities. Table 6 provides a summary of the algorithm’s performance across these demographic variables.

**Table 6.** Summary of heart rate (HR) measurement algorithm performance across different demographic subgroups.

Demographic variables		Reference data points	Measured data points	MAE^a^	DR^b^ (%)
HR (bpm)					
30‐50		671	632	2.36	94.19
50‐70		20,270	17,371	2.12	85.7
70‐90		31,584	26,105	2.67	82.65
90‐110		13,659	11,381	2.68	83.32
110‐130		3591	3054	2.5	85.05
>130		436	365	2.36	83.72
Age (y)					
18‐25		6290	5784	2.35	91.96
25‐35		6706	5716	2.8	85.24
35‐45		11,188	9678	2.46	86.5
45‐55		17,572	14,920	2.21	84.91
≥55		27,967	22,508	2.64	80.48
Sex					
Female		26,466	21,943	2.77	82.91
Male		43,617	36,914	2.33	84.63
BMI (kg/m²)					
<18.5		121	121	1.52	100
18.5‐25		23,791	19,383	2.59	81.47
25‐30		19,569	17,134	2.5	87.56
30‐35		17,882	15,205	2.36	85.03
≥35		8360	6763	2.48	80.9
Race					
Asian American		1122	1076	1.94	95.9
Black or African American		374	337	2.12	90.11
Indian		64,125	53,288	2.52	83.1
Mixed		244	210	2.71	86.07
Unknown or not reported		238	238	1.57	100
White or Caucasian		4108	3759	2.4	91.5
Study participants					
Healthy volunteers		6086	5620	2.27	92.34
ICU^c^ patients		27,194	22,137	2.73	81.40
Patients with apnea		36,931	31,151	2.37	84.35
Comorbidities					
Hypertension		15,184	13,502	2.06	88.92
Diabetes		15,318	13,085	2.46	85.42
Cardiovascular disease		3610	2884	2.6	79.89
Apnea		21,004	17,305	2.68	82.39
Hypothyroidism		3345	2810	2.39	84.01
Trauma		3187	2864	1.73	89.87

a MAE: mean absolute error.

b DR: detection rate.

c ICU: intensive care unit.

The demographic subgroups are as follows:

- HR Range: the algorithm maintained an MAE of 3 bpm or lower across all HR ranges. Notably, in the lower HR range (30‐50 bpm), the algorithm achieved a high DR of 94.19%, while for HRs above 130 bpm, the DR remained robust at 83.72%.
- Age: across all age categories, the algorithm showed strong performance with an MAE consistently below 3 bpm and a DR exceeding 83%, demonstrating its adaptability to different age groups.
- Sex: the algorithm performed equally well for both female and male participants, with MAE values of 2.77 bpm and 2.33 bpm, respectively, and similar DRs (82.91% for females and 84.63% for males).
- BMI: participants with a BMI of less than 18.5 kg m^–²^ achieved an MAE of 1.52 bpm and a DR of 100%, indicating particularly high accuracy in this group. For other BMI categories, the MAE remained below 3 bpm with detection rates above 80%.
- Race: performance was consistently high across racial backgrounds, with an MAE of 2.52 bpm and a DR of 83.1% for Indian participants, while Caucasian and African American groups also demonstrated high accuracy, with DRs exceeding 90%.
- Study participants: the algorithm demonstrated high accuracy across study groups, achieving an MAE of 2.27 bpm and a DR of 92.34% for healthy volunteers, while maintaining strong performance in ICU patients (MAE: 2.73 bpm, DR: 81.40%) and patients with apnea (MAE: 2.37 bpm, DR: 84.35%), showcasing its adaptability to diverse physiological conditions.
- Comorbidity: performance remained consistent across comorbidities, with an MAE below 3 bpm for most conditions. Analysis focused on comorbidities with at least 3000 data points, ensuring robust evaluation. Hypertension (MAE: 2.06 bpm; DR: 88.92%) and diabetes (MAE: 2.46 bpm; DR: 85.42%) showed strong performance, while trauma cases achieved the highest precision (MAE: 1.73 bpm; DR: 89.87%). Cardiovascular disease reported an MAE of 2.6 bpm and a DR of 79.89%, demonstrating reliability even in critical conditions.

These findings confirm the algorithm’s adaptability to diverse population profiles, underscoring its potential as a reliable HR monitoring tool across broad demographic groups in clinical settings.

### Bland-Altman Analysis

**Figure 8.**
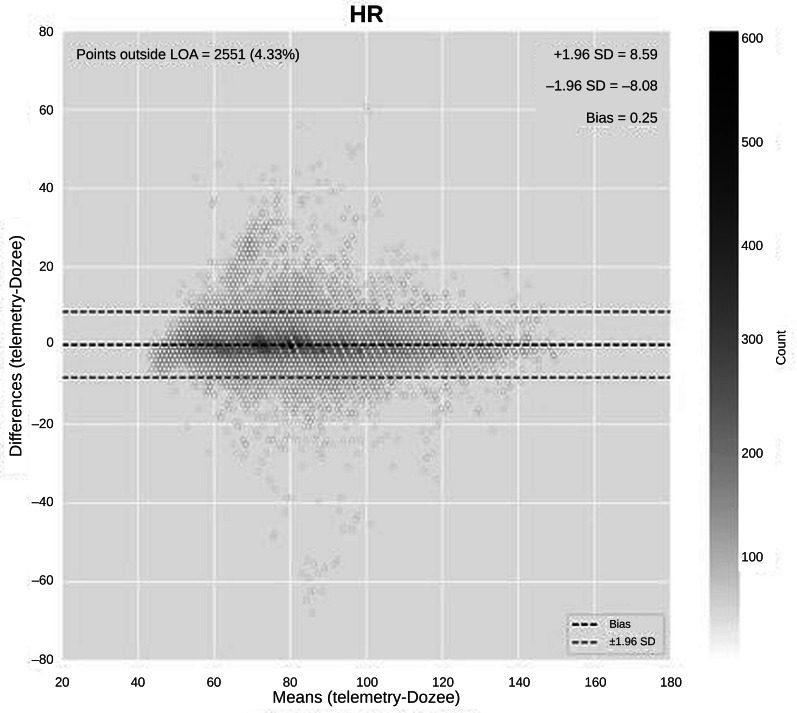
Bland-Altman analysis of the entire testing dataset. HR: heart rate; LOA: limits of agreement.

The Bland-Altman analysis, shown in Figure 8, demonstrates the algorithm’s high accuracy and reliability. The analysis revealed a minimal bias, with a point estimate of 0.25, indicating close alignment with reference HR values. The limits of agreement (LOA), ranging from 8.59 to −8.08, indicate that most differences between the measured and reference values are within 9 bpm, affirming the algorithm’s precision. Notably, 2551 (4.33%) points fall outside the LOA, which represents a small proportion of the total dataset and underscores the algorithm’s strong overall performance. These narrow LOA boundaries, combined with the low bias value, collectively highlight the algorithm’s strong agreement with reference data, confirming its reliability for HR estimation in clinical environments where accuracy is essential.

### Deming Regression and Pearson Correlation Coefficient Analysis

**Figure 9.**
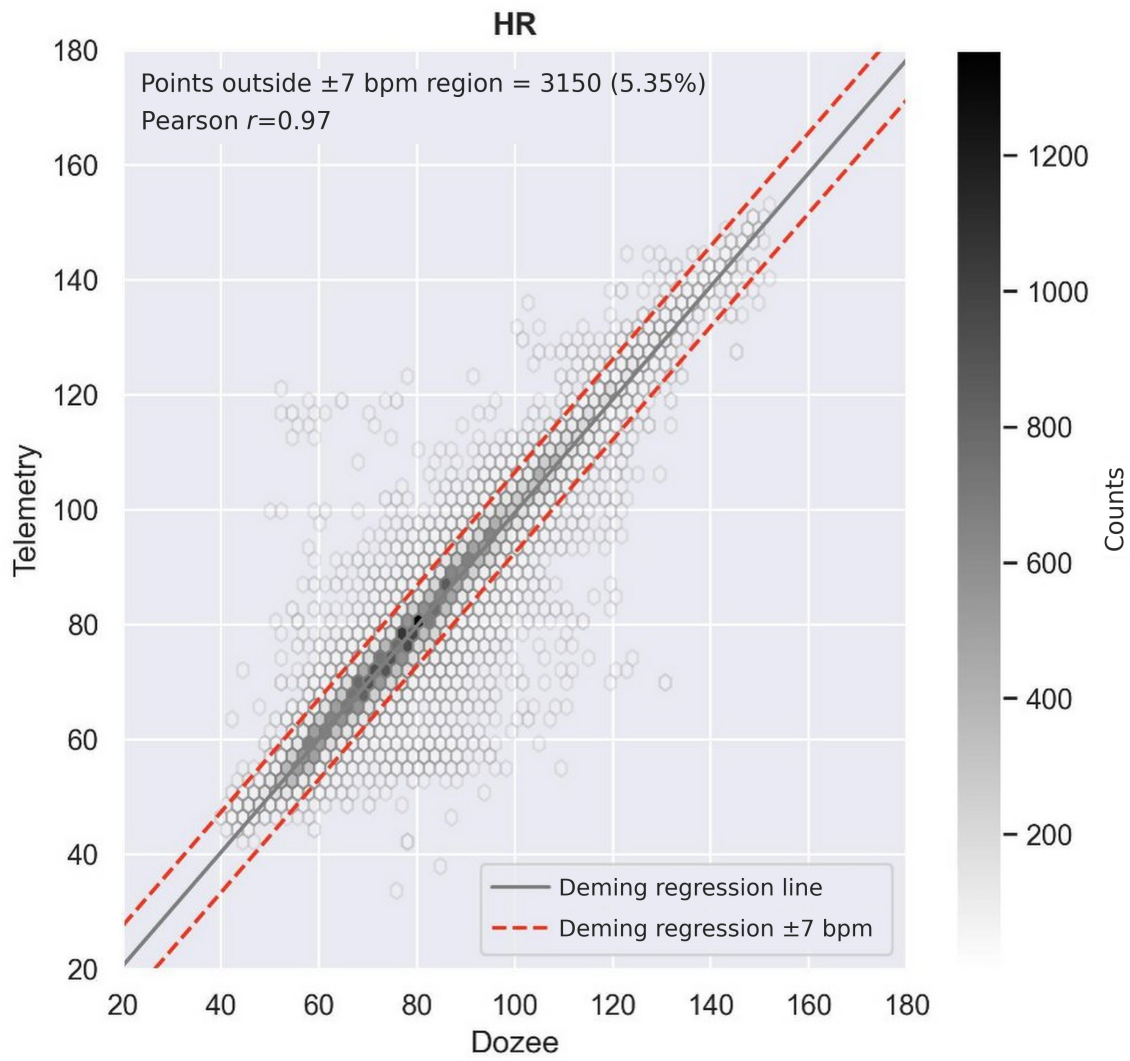
Deming regression with the Pearson coefficient for the entire testing dataset. HR: heart rate.

The Deming regression analysis, complemented by the Pearson correlation coefficient, provided further evidence of the algorithm’s alignment with reference HR values. Figure 9 illustrates the linear relationship between the algorithm’s HR measurements and the clinical reference values. With a Pearson correlation coefficient of 0.97, the algorithm demonstrated a strong correlation with the reference HR data, reinforcing its accuracy and reliability. Notably, 3150 (5.35%) points fall outside the ±7 bpm region, further highlighting the precision of the algorithm’s measurements. The high correlation, together with minimal observed bias, underscores the algorithm’s potential for consistent and accurate HR monitoring in diverse clinical settings. These findings validate the algorithm’s performance, confirming its suitability for contactless patient monitoring applications that require reliable HR data.

## Discussion

### Advancements in Contactless HR Monitoring

This study introduces a highly accurate, contactless HR measurement algorithm based on BCG signals, specifically designed for continuous, noninvasive monitoring in clinical settings. By combining adaptive signal processing techniques with ML-based peak detection, the algorithm effectively addresses challenges in BCG monitoring, such as noise, motion artifacts, and physiological variability. Unlike traditional methods that rely on fixed filtering and thresholding, this algorithm dynamically suppresses noise while preserving HR-related features, ensuring reliable performance under nonstationary conditions caused by patient movement and physiological fluctuations. The algorithm achieved an MAE within 3 bpm and a DR above 80% across diverse settings, demonstrating reliable HR monitoring despite motion artifacts and physiological variability.

A significant strength of this algorithm is its adaptability across demographic subgroups, including age, sex, BMI, and race. This versatility enhances its applicability in dynamic, real-world environments. The CNN architecture represents a substantial advancement over conventional threshold-based methods, which can obscure true cardiac peaks in noisy settings. By maintaining independent datasets for training, tuning, and testing, we minimized the risk of overfitting, ensuring the algorithm generalizes well across different environments. This careful design enables the algorithm to consistently deliver reliable HR measurements, making it suitable for hospital wards and potential applications in home and remote monitoring.

To our knowledge, this is the first BCG-based HR monitoring study to combine time-domain and frequency-domain processing with a deep learning model and to validate it across such a broad range of conditions. Our CNN-based algorithm demonstrates notable improvements in accuracy, robustness, and adaptability compared to existing BCG-based HR measurement solutions. With an MAE of 3 bpm, our algorithm surpasses the accuracy of some commercial contactless solutions, such as EarlySense, which reports an MAE of 5 bpm, setting a new benchmark in contactless HR monitoring. Few studies report comparable MAE values. For example, Vesterinen et al [28] assessed the Emfit QS, a commercial contact-free BCG device, achieving an MAE of 2.4 bpm but with a higher rate of missing data compared to ECG. Similarly, Clemente et al [29] used a bed-mounted sensor system with an MAE of 2.41 bpm, while Proll et al [30] achieved an average MAE of 2.07 bpm across a smaller sample size of 14 patients. Jung et al [31] tested a multichannel load cell bed, achieving an MAE of 1.76 bpm in the supine posture, with a maximum error of 3.03 bpm in lateral postures. Although these solutions show promising accuracy, their limited validation in diverse clinical environments restricts generalizability.

The Bland-Altman analysis underscores the algorithm’s high accuracy and reliability, validating its potential for clinical application. The minimal bias observed indicates a close alignment between the algorithm’s HR measurements and the reference values, highlighting its precision and robustness under diverse conditions. The LOA show that most differences between measured and reference HR values fall within a clinically acceptable range, with only a small fraction of data points outside these limits. This reinforces the algorithm’s reliability and strong agreement with reference data, even in the face of physiological and environmental variability. Notably, the bias and LOA observed in this analysis are comparable to those reported for HR measurements using BCG under real-world conditions. For instance, studies using BCG-based technologies, such as EarlySense in postoperative settings and Emfit in sleep lab environments [32,33], have demonstrated similar bias (−1.4 and 1.79) and LOA (10.4 to −13.2 and 19.11 to −15.33), further validating the robustness and reliability of the algorithm in practical, uncontrolled scenarios. This alignment with real-world performance benchmarks emphasizes the algorithm’s suitability for clinical applications where accuracy and consistency are paramount.

The Deming regression analysis, supported by the Pearson correlation, highlights the strong alignment between the algorithm’s HR measurements and the reference standard. The analysis demonstrates a robust linear relationship, reflecting the algorithm’s precision and reliability across diverse conditions. This strong correlation is particularly important in clinical settings, where accurate trend detection and reliable HR measurements are essential for timely interventions and effective patient management. By ensuring minimal variability and maintaining close agreement with reference values, the algorithm enables health care providers to monitor physiological changes confidently. The findings align with those of other noninvasive HR monitoring systems [34], further validating the algorithm’s performance. This establishes the algorithm as a viable tool for both hospital and remote monitoring, offering a reliable solution for continuous, contactless HR monitoring to improve patient care and outcomes.

Many existing approaches to HR estimation exhibit limitations in sample diversity and applicable HR ranges, which can impact their generalizability in clinical practice. For instance, Sumali et al [35] used a CNN algorithm for peak detection but limited their analysis to an HR range of 60 to 100 bpm, potentially overlooking extreme HRs encountered in clinical practice. Feng et al [36] used STFT and empirical mode decomposition for peak extraction on a small sample of 10 healthy participants and 2 patients with coronary heart disease, which limited applicability due to narrow demographics. Pino et al [37] tested only 34 normal participants and 24 with atrial fibrillation in controlled settings, which restricts the generalizability of their findings. Moukadem et al [38] based their analysis on data from just 3 participants, further limiting the applicability of their results. Yang et al [22] used peak detection with discrete wavelet transform across a small dataset of 24 participants, with recordings lasting about 3 minutes; this restricted duration and participant diversity limit applicability. Schranz et al [39] introduced a supervised deep learning method for J-peak extraction from BCG measurements. By modeling discrete heartbeat events with a symmetric, continuous kernel function (surrogate signal), this approach enhances target heartbeat detection. Their evaluation is based on a BCG dataset from 11 participants, collected over 17 nights of sleep, totaling 134 hours of ECG reference data that investigated various deep learning architectures, including convolutional, recurrent, and hybrid models, on a held-out testing set of 14 patients [37,39]. Choe et al [40] proposed an advanced algorithm using a moving dispersion calculation method to accurately identify heartbeat locations. This is enhanced by an adaptive peak detection technique that dynamically adjusts the detection window based on predicted heartbeat locations. The method effectively operates across a HR range of 40 to 110 bpm. In contrast to the above-mentioned studies, our algorithm stands out due to its validation across a broad HR range and diverse patient demographics, enhancing robustness and generalizability for real-world applications [40].

Currently, patients in hospital wards are often monitored solely through spot checks [41], highlighting the need for continuous HR monitoring. In this context, an error margin of 3 bpm is deemed acceptable for several reasons. This margin emphasizes trend detection over precise measurements, enabling health care providers to identify significant changes in HR that may indicate patient deterioration or response to treatment. Recognizing the natural fluctuations in HR due to physiological variability, this margin accommodates normal variations while still delivering clinically relevant data. Furthermore, it has been reported in the literature that HR is deemed clinically acceptable if it aligns within ±5 bpm or within ±10% of the reference standard, supporting the adequacy of these thresholds. Moreover, previous studies validate the adequacy of a ±3 bpm for effective monitoring, reinforcing its appropriateness for enhancing patient management and outcomes [3,42].

The key strength of this study lies in its innovative dual-domain algorithm, which integrates time-domain HR peak detection with STFT analysis. While the STFT effectively captures the fundamental heart-rate frequencies, the CNN adds significant value by identifying complex temporal and spectral patterns that may not be apparent in traditional frequency-domain representations and by improving robustness to noise and artifacts through learned pattern recognition. This is particularly advantageous in real-world clinical settings, where physiological variability and movement-related disturbances are common. This dual-domain CNN approach addresses the noise or artifact challenges noted in earlier methods and elevates the performance to a level suitable for demanding health care environments.

By combining these advanced techniques, our algorithm sets a new benchmark in contactless HR monitoring. Rigorously trained on a large and diverse dataset that includes varying levels of motion artifacts and physiological diversity, particularly from ICU settings with inherent noise and HR fluctuation, this algorithm ensures reliable HR detection across a wide range of clinical scenarios, positioning it as a transformative solution for HR monitoring in challenging health care environments. Importantly, we validated the algorithm in both controlled and real-world environments, demonstrating practical robustness. Performance remains consistent across age, sex, BMI, ethnicity, and comorbidities, underscoring its strong generalizability.

While this study validates the algorithm effectively, several limitations must be considered. The BCG data used for training and validation were exclusively collected from piezoelectric sensors, which may limit the algorithm’s applicability to other sensor types and reduce its generalizability. Testing across various BCG sensor technologies is recommended to enhance adaptability. Furthermore, while the algorithm performs well across a broad HR range, its accuracy with highly irregular heart rhythms, such as severe arrhythmias, remains untested and may require tailored adaptations. The CNN-based architecture also introduces computational demands that could hinder real-time processing in resource-limited environments; optimizing the algorithm for lower computational needs could improve scalability for telemedicine and home care applications. Additionally, we acknowledge a limitation in methodological transparency: the full CNN architectural details and certain parameter values are not disclosed due to the proprietary nature of the algorithm. This constraint, while necessary from an intellectual property standpoint, means that external researchers cannot yet fully reproduce the model. We have mitigated this by rigorously validating the algorithm on independent datasets. Finally, the algorithm’s performance is influenced by the characteristics of the training data, potentially affecting generalization to demographics or conditions that are underrepresented in the dataset.

Future studies could further enhance the algorithm by integrating additional vital signs, such as respiratory rate, to create a more comprehensive monitoring system. Expanding the dataset to include more data points for comorbidities will provide a stronger foundation for these refinements. Investigating the impact of comorbidities on BCG signals could further optimize the algorithm for specific patient populations, enabling customization for individual health profiles. As the demand for contactless patient monitoring grows, ongoing updates and algorithm refinements based on real-world data, combined with enriched datasets, will be essential to maintaining the algorithm’s accuracy, adaptability, and relevance in clinical practice.

### Conclusion

In summary, this study presents a novel CNN-based, contactless HR measurement algorithm using BCG signals, designed for reliable, continuous HR monitoring in diverse clinical environments. By integrating advanced signal processing with ML, this approach achieves a high degree of accuracy, adaptability, and robustness, establishing a new benchmark for noninvasive monitoring solutions. The algorithm’s capacity to perform reliably across varied demographics and conditions highlights its potential as a transformative tool for real-time HR monitoring, promising improved patient outcomes through timely detection and interventions. Future research will focus on expanding the algorithm’s validation across various clinical environments and integrating additional vital signs to create a more holistic and effective monitoring solution.

## Abbreviations

BCG: ballistocardiography
CNN: convolutional neural network
DR: detection rate
ECG: electrocardiography
FDA: Food and Drug Administration
HR: heart rate
ICU: intensive care unit
LOA: limits of agreement
MAE: mean absolute error
ML: machine learning
STFT: short-time Fourier transform
